# Complete Prevention of Bubbles in a PDMS-Based Digital PCR Chip with a Multifunction Cavity

**DOI:** 10.3390/bios14030114

**Published:** 2024-02-21

**Authors:** Shiyuan Gao, Tiegang Xu, Lei Wu, Xiaoyue Zhu, Xuefeng Wang, Ying Chen, Gang Li, Xinxin Li

**Affiliations:** 1 State Key Laboratory of Transducer Technology, Shanghai Institute of Microsystem and Information Technology, Chinese Academy of Sciences, Shanghai 200050, China; gaoshy1@shanghaitech.edu.cn (S.G.); chenying@mail.sim.ac.cn (Y.C.); 2 College of Materials Science and Opto-Electronic Technology, University of Chinese Academy of Sciences, Beijing 100049, China; 3 School of Information Science and Technology, ShanghaiTech University, Shanghai 201210, China; 4 Metabolomics Center, Haixia Institute of Science and Technology, School of Future Technology, Fujian Agriculture and Forestry University, Fuzhou 350002, China; xiaoyuezhu@fafu.edu.cn; 5 Key Laboratory of Optoelectronic Technology and Systems, Ministry of Education, Defense Key Disciplines Lab of Novel Micro-Nano Devices and System Technology, Chongqing University, Chongqing 400044, China; gang_li@cqu.edu.cn

**Keywords:** digital PCR chip, polydimethylsiloxane, multifunction cavity, water loss, bubble generation, parylene C shell, negative pressure environment, saturated vapor pressure of water

## Abstract

In a chamber-based digital PCR (dPCR) chip fabricated with polydimethylsiloxane (PDMS), bubble generation in the chambers at high temperatures is a critical issue. Here, we found that the main reason for bubble formation in PDMS chips is the too-high saturated vapor pressure of water at an elevated temperature. The bubbles should be completely prevented by reducing the initial pressure of the system to under 13.6 kPa to eliminate the effects of increased-pressure water vapor. Then, a cavity was designed and fabricated above the PCR reaction layer, and Parylene C was used as a shell covering the chip. The cavity was used for the negative generator in sample loading, PDMS degassing, PCR solution degassing in the digitization process and water storage in the thermal reaction process. The analysis was confirmed and finally achieved a desirable bubble-free, fast-digitization, valve-free and no-tubing connection dPCR.

## 1. Introduction

Polymerase chain reaction (PCR) is a modern technology with which to clone DNA by exponentially increasing nucleic acid double strands through cycles of denaturing, annealing and extension [1]. A variety of PCR methods such as reverse transcription PCR [2], multiplex PCR [3], NEST PCR [4], and quantitative PCR [5], commonly known as second-generation PCR technology, have been widely used in molecular biology, gene engineering, gene detection, biochemical, clinical analysis, diagnosis and disease prevention [6]. A third-generation PCR technology, digital PCR (dPCR), springs up for detection sensitivity of a significantly elevated power, enabling the direct quantitative analysis of absolute DNA concentrations and low-abundance samples [7,8].

The principle of dPCR is based on the statistics of the Poisson distribution. In a typical dPCR experiment, the sample is diluted and divided into a large number of separate reaction units, so that each reaction unit contains either a few or zero copies of the target DNA molecule. The ratio of positive reactions to negative reactions can be used to directly quantify the clonally amplified nucleic acids strands [9]. Since a larger number of reactions and a smaller reaction unit volume would contribute to the accuracy of dPCR results, the microfluidic system, which has the advantages such as high throughput, low sample and reagent consumption, and precise and massive sample partition, nicely caters to the needs of dPCR development. The microfluidic system also introduces additional benefits such as small thermal mass, low thermal inertia, and rapid heat transfer, which leads to efficient detection and low cost [10,11].

In chamber-based dPCR (cdPCR) platforms, the chambers are precisely fabricated into fine structures with a defined volume of reaction units, avoiding nonuniformity in reaction unit size. Additionally, the regular arrays of the chambers are convenient for sample loading, partition and fluorescence signal reading [12]. The throughput of cdPCR has reached 440,000 cm^−2^ of reactors and greater-than-megapixel reaction units per chip [13].

PDMS is a popular material for many microfluidic systems due to its properties including transparency, chemical resistance, easy fabrication and low cost [14,15]. Moreover, its good gas permeability is essential for dead-end sample loading with positive pressure [13], negative pressure [16,17] or self-priming with pre-degassed PDMS bulk [18,19]. However, the air molecules in PDMS nanopores will cause water evaporation and bubble generation during PCR thermal cycles [20,21,22,23,24,25,26]. An embedded layer of low-permeability polymer such as parylene C [13] and fluorosilane polymer [27] covering just above the digital PCR array have been shown to reduce water loss. However, these methods cannot fully prevent water evaporation so hydration channels are still needed to compensate for water loss. In addition, the embedded polymer will lead to a decreased sample loading rate. We managed to solve the issue of water evaporation by fabricating a cavity above the reaction chamber to accelerate sample loading and filling the cavity with water to replenish water molecules lost from the sample [28]. However, this cannot fully solve the bubble generation problem. The existence of a small amount of unequal-size bubbles in the reaction chambers could result in an inaccurate reaction volume. Bubbles can also push the PCR sample out of the chip or break the isolation barrier between the reaction units to cause cross contamination [21]. Micro PDMS valves [29] have often been used to prevent bubbles from appearing, but the micro valves must be connected to the pressure control tubing throughout the reaction. We also prevented bubble generation by sealing the chip with thermally curable oil [30] or providing an external liquid-phase high-pressure environment [31]. However, introducing viscous oils into microchannels is time-consuming and the high-pressure environment of the external liquid phase requires specialized instrumentation. Overall, sample loading, partitioning, water loss, bubble generation and time consumption all need to be optimized for a cdPCR chip fabricated with PDMS.

## 2. Mechanism Analysis for Bubble Generation

**Figure 1:**
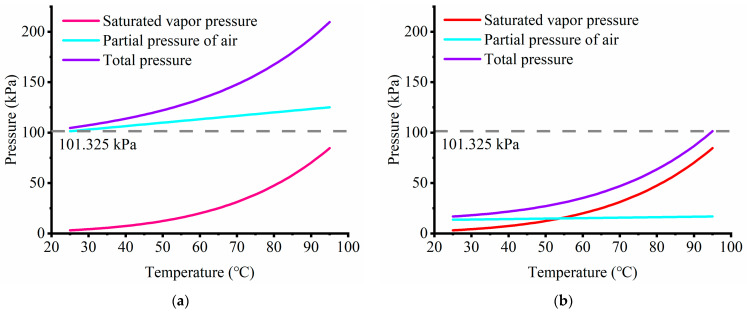
Relation of pressure versus temperature in the PDMS nanopore: (**a**) 101.325 kPa (standard atmospheric pressure) initial pressure; (**b**) 13.602 kPa initial pressure.

In this work, we first analyzed the mechanism of bubble formation. The maximum temperature for digital PCR thermal cycling is often 95 °C. As the temperature rises, more water vaporizes; thus, the saturated vapor pressure of water increases significantly, which in turn increases the air pressure inside the PDMS nanopores and results in bubble formation when a chip is heated from 25 °C (room temperature) to 95 °C. Actually, the main component of the bubble is not the dry air molecules but the gaseous water molecules, which is undervalued in previous studies. Thus, the total pressure of gas will increase even more with an increase in the partial pressure of water vapor. On the other hand, the saturated vapor pressure of water increases exponentially with increasing temperature. As a result, the gas pressure inside the chip increases rapidly during temperature elevation and causes bubble formation due to the effect of the water vapor (Figure 1a).

On the other hand, although a saturated vapor pressure of water cannot be changed at a fixed temperature, we can lower the partial pressure of air to reduce the total pressure of the system. In order to prevent the formation of air bubbles inside the chip, the pressure of the air inside the chip must be reduced to be lower than the outside atmospheric pressure during thermal cycling. When the temperature increases from 25 °C to 95 °C, the saturation vapor pressure of water increases from 3.169 kPa to 84.529 kPa. According to Dalton’s law of partial pressure, the partial pressure of dry air can be expressed as follows:

(1) $$p_{A}=p-p_{WV}$$

where *p_A_* is the partial pressure of the dry air, *p* is the total pressure of the gas, and *p_WV_* is the saturated vapor pressure of water. Therefore, the partial pressure of dry air at 95 °C must be less than 16.796 kPa to ensure that no air bubbles are formed. The maximum partial pressure of dry air at 25 °C can be calculated according to Charles’s law:

(2) $$\frac{p_{2}}{p_{1}}=\frac{T_{2}}{T_{1}}$$

where *p*_1_ and *p*_2_ are the partial pressures of dry air at *T*_1_ (25 °C, 298.15 K) and *T*_2_ (95 °C, 368.15 K), respectively. Therefore, the maximum air partial pressure inside the chip at 25 °C should be 13.602 kPa. If the pressure in the system is lower than this value, and both the air pressure after thermal expansion and the pressure of water vapor in the PDMS nanopore are lower than the atmospheric pressure, bubbles will not form during the temperature elevation phase of PCR (Figure 1b).

## 3. Design and Modeling

### 3.1. Chip Design and Principle

**Figure 2:**
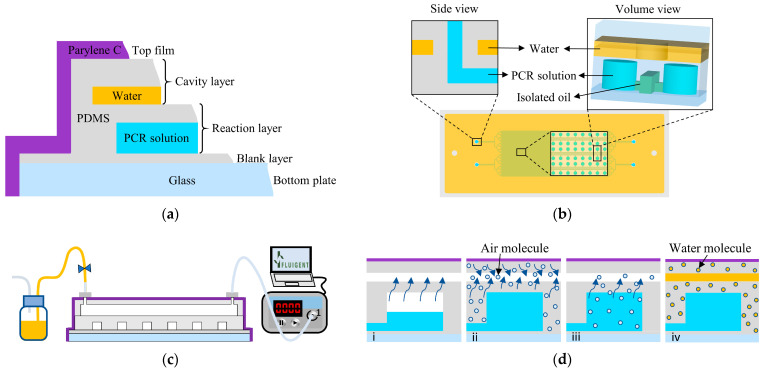
The digital PCR system. (**a**) Schematic diagram of the layered device structure of the microfluidic chip, which is composed of five layers: (i) a glass substrate, (ii) a blank layer of PDMS spun on the glass slide, (iii) a layer of PDMS for the PCR reaction bonded on the blank PDMS layer, (iv) a layer of PDMS for the cavity that was bonded on the reaction layer, and (v) a layer of parylene C coated around the chip. The reaction layer contains PCR chambers and sample channels. The cavity layer covers the reaction layer and carries the pillar to prevent collapse. (**b**) The overlay structure and a zoomed-in view of the dPCR chip. The cavity covers the whole sample area except for the inlet, outlet and edge of the chip. (**c**) The utilization of the soft tube valve during degassing and water filling processes to maintain a constant negative pressure within the cavity. (**d**) Four functions of the cavity: (**i**) sample solution loading, (**ii**) PDMS degassing, (**iii**) PCR solution degassing, and (**iv**) water molecule compensation.

In order to reduce the initial partial pressure of the air in the chip, a sandwich structure device comprising a parylene C cover, a three-layer PDMS chip, and a glass-bottom slide (Figure 2a,b) was designed. The middle layer of the PDMS chip is a PCR reaction layer that contains microscale reaction chambers and connecting channels. The top layer of the PDMS chip contains a cavity with an array of pillars for structural support. The cavity is used for sample loading and PDMS degassing. Furthermore, the cavity covers up to the edge of the chip except for the inlet and outlet to ensure that air in the nanopores of the PDMS can be quickly evacuated in the whole chip and to allow the establishment of a safe and secure negative pressure environment. The bottom blank PDMS is used to seal the PCR chambers and channels of the reaction layer.

To prevent the occurrence of bubbles, it is necessary to maintain negative pressure in the PDMS. Therefore, when filling the cavity with water, the inlet valve of the cavity was slowly opened while maintaining a low flow rate, ensuring that the negative pressure in the cavity remained stable (see Figure 2c). However, some small-volume dead ends in the cavity were inevitable. To fill these areas, the negative pressure stored inside the PDMS was used to drive water into them. Owing to PDMS’s distinct air and water barrier function, the air enclosed within it remained at a negative pressure even when the soft tubes of the cavity were unplugged.

As shown in Figure 2d, the cavity contains four functions: (i) Negative pressure applied to the cavity draws air out of the reaction chamber and causes a rapid reduction in pressure in the whole system, which causes a pressure drop between the inlet and reaction chamber to drive sample loading. (ii) PDMS is a mesoporous material and a large amount of air is absorbed in the pores. The concentration of air molecules in the PDMS is higher than that of molecules in the cavity under negative pressure, so the absorbed air molecules in the PDMS will diffuse outwardly to degas the PDMS. (iii) This negative pressure also induced gaseous substances to escape from the solution into the PDMS because the solubility of gas reduces with pressure and the dimension of the channel is very small. (iv) Filling the cavity with water can create a moisture environment so that the water molecules from the cavity will diffuse into the PDMS to reduce the loss of the sample solution.

### 3.2. Chamber Filling and System Degassing Analysis

The PDMS material can absorb gas and allow gas to diffuse even when in a solid state. Permeability, solubility, and diffusivity data and equations for PDMS have been widely reported [32]. Thus, it is possible to calculate the concentration distribution of the gas in the PDMS during degassing, the sample loading time by degassing, and the time required for the PDMS to reach a specific pressure by pumping.

**Figure 3:**
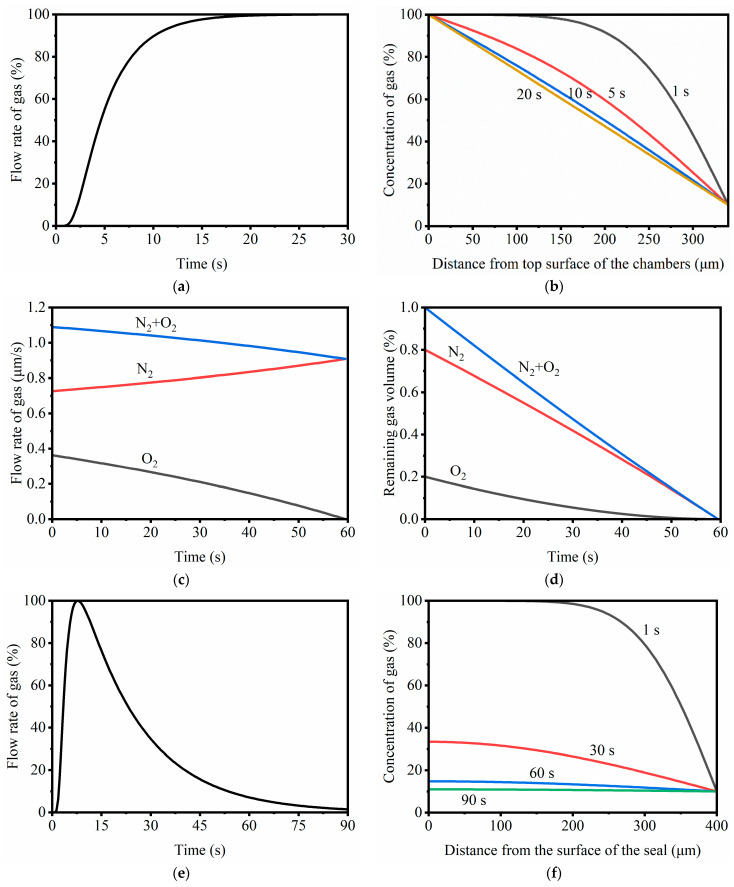
(**a**) The gas flow rate on the chamber surface increasing with time. (**b**) The concentration distribution of air in PDMS membrane from chamber surface to cavity surface. (**c**) The linear flow rate of the gas passing through the PDMS. (**d**) The remaining gas volume in the chamber. (**e**) The degassing rate of the PDMS surface sealed with parylene C or glass. (**f**) The gas concentration in the PDMS membrane from the sealed surface to the cavity surface.

The concentration of gases in PDMS roughly exhibits a linear relationship with the pressure at a fixed temperature. At 1 atm, the air concentration in PDMS is 0.11 cm^3^ (standard temperature and pressure, STP)/cm^3^, while it is 0.011 cm^3^ (STP)/cm^3^ on the cavity surface when the pressure in the cavity is 0.1 atm [33]. In dead-end chamber loading, the gas in the chambers passes through the PDMS membrane and flows into the vacuum cavity, directing the PCR solution from the channels into the chambers. The concentration profile, *C*(*x*, *t*), in the PDMS membrane can be calculated via Fick’s second law of diffusion:

(3) $$\frac{\partial C}{\partial t}=D\frac{\partial^{2}C}{\partial x^{2}}$$

where *D* is the diffusivity (*D_air_* = 3.4 × 10^−6^ cm^2^ s^−1^), *x* is the location, and *t* is the time. The thickness of the PDMS film between the chamber and the cavity is 340 μm. The numerical calculation shows that the air flow rate (*∂C*/*∂t*) at the surface of the chamber (*x* = 0) reaches 90% of its maximum in 10 s (Figure 3a). The gas in the PDMS forms a stable concentration gradient and reaches its maximum flow rate in less than 20 s (Figure 3b). This result indicates that the amount of time to build a constant flow is short.

The permeability of a polymer membrane by a pure penetrant is given by

(4) $$P=\frac{Nl}{p_{2}-p_{1}}$$

where *P* is the gas permeability coefficient, *N* is the gas flux, *p*_2_ is the pressure in the chamber (1 atm), *p*_1_ is the pressure in the cavity (0.1 atm), and *l* is the PDMS membrane thickness (340 μm). For steady-state flux across a thin membrane, the volumetric flow rate can be determined by

(5) $$\frac{dV}{dt}=\frac{PA(p_{2}-p_{1})}{l}\frac{T}{273}\frac{76}{p_{atm}}$$

where *dV*/*dt* is the volumetric flow rate in cm^3^ s^−1^, *A* is the surface area, *T* is the absolute temperature in kelvin, and *p_atm_* is the atmospheric pressure (1 atm, 76 cm Hg). Thus, the steady-state gas flow rate across the thin PDMS membrane is linearly proportional to total pressure difference across the PDMS membrane and inversely proportional to the PDMS membrane thickness. To simplify the analysis, we assume that he gas only transports from the top surface of the chambers. The rising speed of liquid level is given by

(6) $$r=\frac{dV}{dtA}=\frac{P(p_{2}-p_{1})}{l}\frac{T}{273}$$

where *r* is the linear rising speed of liquid level in cm s^−1^. We also assume that the N_2_ and O_2_ in air began with a ratio of 4:1 and that the partial pressure in chamber was proportional to that of the remaining amount of these two gases. Permeability coefficients of N_2_ and O_2_ in PDMS at 35 °C are 400 Barrer and 800 Barrer (1 Barrer = 10^−10^ cm^3^ (STP) cm/(cm^2^ s cm Hg)), respectively. Then, the O_2_ flow rate, N_2_ flow rate, and air flow rate of chamber filling can be calculated in accordance with Equation (6), and the gas flow rates decrease with time (Figure 3c). The height of the PCR chambers is 60 μm and the chambers will be filled in 60 s (Figure 3d). It should be noted that flows from the side wall will accelerate sample loading, which means that the filling process can be finished quickly with the method.

The degassing cavity is no more than 400 μm from the surface of the PDMS that is sealed with glass or parylene C. Then, gas the concentration and degassing rate are also calculated in accordance with Equation (3) (Figure 3e,f). As a result, PDMS takes around 90 s to reach 10 kPa.

### 3.3. Stability of the PCR Solution Compensated for with the Water Cavity

The top and bottom surface of the chip are sealed with the parylene layer and glass, respectively; therefore, very little water can flux out of the chip during thermal cycling. The maximum fractional loss of water is from PDMS absorption. Though PDMS is a hydrophobic material, water vapor still has the ability to diffuse into the bulk PDMS and be absorbed through the PDMS. The water in the chip could be evaporated into gas phase using heat, and the saturated vapor pressure of water is increased with temperature. The thermal cycling of PCR is at 95 °C, 60 °C and 72 °C; therefore, 70 °C is selected for approximate calculation. The minimum thickness of the PDMS is set to be 400 μm and the size of the PDMS membrane is larger than that of the chamber array to avoid difficulties in fabrication, membrane transfer, multilayer layer bonding, and accommodating external channels and ports.

Since the volume of the PDMS is about 1.12 cm^3^ (2 cm × 7 cm × 0.08 cm), and the saturated water vapor sorption through PDMS at 70 °C is about 1.4 cm^3^ (STP)/g, the density of water vapor (STP) is 0.80 mg/cm^3^, and the density of PDMS is 1.85 g/cm^3^; therefore, a 1.12 cm^3^ PDMS is capable of adsorbing up to 2.32 mg of water. In the situation without the cavity layer, 0.151 nL of water will be lost from each chamber. Since 15360 PCR chambers are in each chip, the PCR solution will be lost heavily considering that the volume of the chamber is only 0.47 nL. With the cavity layer, the problem of water loss is easily solved. Water vapor will diffuse into the PDMS both from the cavity and the chambers. Due to location differences, water vapor in the chambers could diffuse into a little space into the PDMS just around the chamber. Furthermore, water vapor in the cavity will diffuse up into the bulk PDMS or down to the PDMS around the chamber. Most of the water is lost from the cavity and only a little amount of water is lost from the chambers. Water loss from the chamber can be compensated for with a high vapor pressure of water in the cavity during thermal reaction. Moreover, since the water in the cavity is about 29.56 mg (1.6 cm × 6.6 cm × 0.004 cm, deducting 30% used for supporting pillars), 2.32 mg of water loss into the bulk PDMS is very little and can be ignored.

## 4. Experiment

### 4.1. Chip Fabrication

The length of the chip is 7 cm and the width is 2 cm. The cylindrical microchambers in the reaction layer have a height of 60 μm and a diameter of 100 μm. Rows of chambers are connected to the main channels (height = 60 μm, width = 60 μm) through branch channels (height = 10 μm, width = 20 μm). The diameter of the support columns contained in the cavity layer is 100 μm. The volume of each reaction unit is 0.47 nL. Each chip contains 15360 individual reaction units.

The chips are fabricated using a multilayer soft lithography technique. The detailed manufacturing process is shown in Figure S1. All molds were fabricated on 4-inch silicon wafers. The chip patterns were designed using AutoCAD and printed on transparency films by a high-resolution printer. The mold of the cavity layer was fabricated using SU8-3050 photoresist (Microchem) to deposit a 40 μm high cavity and pillar features. The mold of the reaction layer was fabricated with two lithographic steps. First, 10 μm high branch channels were fabricated using SU8-3005 photoresist. Then, 60 μm high chambers and fluidic channels were constructed using SU8-3050 photoresist. All the photoresist processing was performed in accordance with the manufacturer’s specifications. Then, both of the molds were deposited with 200 nm parylene C to prevent the adhesion of PDMS.

The cavity layer is 400 μm in thickness for mechanical stability to allow reliable use and was created by pouring a mixture of 10 parts of the PDMS prepolymer and 1 part of the cure agent into the reaction mold. After baking on a hotplate at 80 °C for 30 min, the PDMS block on the mold was peeled off and we punched out two holes for the cavity layer. Then, an 400 μm thick PDMS reaction layer was created using the same method. Next, the 400 μm thick cavity PDMS layer was aligned and bonded to the reaction layer under home-made alignment equipment after being activated with oxygen plasma. After being baked at 80 °C for 2 h on a hotplate to enhance the bonding strength, the two bonded layers were peeled off from the mold of the reaction and we punched out inlets and outlets of the PCR solution. Then, the blank PDMS layer was generated by spin coating PDMS on a glass slide at 3000 rpm for 1min and curing it at 80 °C for 15 min. The blank layer on the slide was bonded with the top two layers of PDMS too and baked at 80 °C overnight. Finally, the chip was prepared after deposited coating with 4 μm of parylene C (Coating application system Parylene P6, Diener electronic GmbH & Co. KG, Ebhausen, Germany).

### 4.2. Temperature Control Instruments for Real-Time Observation under Microscope

During the heating process, the solution in the chamber and the cavity may evaporate and generate bubbles. To observe the evaporation of the solution and the formation of bubbles in real time under a microscope, a small, transparent and controllable heating system was constructed. The system consists of a home-made temperature controller, a piece of indium–tin oxide (ITO)-coated glass and a Pt100 temperature sensor (Figure S2). The ITO coated glass was used as a heater. The temperature sensor Pt100 was glued tightly onto the surface of the ITO glass to the detection temperature in real time. Temperature was adjusted with the temperature controller by regulating the output of the power source according to the feedback temperature from the Pt100 sensor. The PDMS chip was taped to the surface of ITO glass opposite to the Pt100 sensor, and the Pt100 sensor was placed under the central area of the dPCR chip for accurate temperature detection. The temperature of the ITO glass was set up with a computer.

### 4.3. PCR Reaction

A template synthesized based on epidermal growth factor receptor (EGFR) gene sequences (Supplementary Materials, Template sequence, 214 nt) was used for dPCR amplification. Primers and probes were designed with the Primer premier5 software. A pair of primers could amplify a 194 bp product of EGFR exon 19 (forward primer: 5′-ATCCCAGAAGGTGAGAAAGT-3′; reverse primer: 5′-TGTGGAGATGAGCAGGGTCT-3′). A FAM labeled MGB hydrolysis probe (5′FAM-AAGCCAACAAGGAAATC-MGB 3′) was used to trace all the PCR products. Template forward/reverse primers and probes were synthesized by Invitrogen. All DNA reagents had to be stored at −20 °C prior to use. The PCR mixture in a total volume of 10 μL contained 5 μL of 2 × Light Cycler^®^480 Probe Master (Roche), 400 nM of the forward and reverse primer each, a 250 nM MGB probe, and a 1 μL template. Before sample loading, all PCR mixture components including the DNA template, PCR master mix, forward/reverse primers, and probes needed to be mixed in advance.

After sample loading and partition, system degassing and water filling into the cavity, the UV glue was then added to the inlet and outlet ports, which isolated the outside air with a parylene C shell from the chip. Thermal cycling reaction was performed using an in situ PCR instrument (Mastercycler nexus flat, Eppendorf, Germany) with two-step PCR thermocycling. First of all, the chip was heated for 10 min at 95 °C to activate Fast Start Taq polymerase. Then, forty thermal cycles of 95 °C for 20 s and of 60 °C for 25 s were then performed to amplify the target DNA, which took about 50 min in total.

### 4.4. Data Acquisition and Analysis

To verify the performance of the dPCR chip, the template was diluted into a series of concentrations including 4.7 copies/μL, 4.7 × 10^1^ copies/μL, 4.7 × 10^2^ copies/μL and 4.7 × 10^3^ copies/μL. The concentrations of the template DNA in the dPCR chip were calculated according to the Poisson statistics principle, as shown in the following equation:

(7) $$c=-\frac{ln(1-\frac{m}{n})}{V}$$

where *n* is the total number of chambers in each dPCR chip, *m* is the number of positive chambers, *m*/*n* is the fraction of positive chambers, and V is the chamber volume. Because the volume of each chamber is 0.47 nL, 1 copy/chamber = 2.13 × 10^3^ copies/μL. All experiments were repeated three times. The standard deviation formula was used to calculate the error in three replicate experiments.

Bright-field images and enlarged fluorescent images are acquired with a stereo microscope (Olympus) by the Image ProPlus V 6.0 software. The fluorescent images of the chip after amplification are acquired using Maestro Ex IN-VIVO Imaging System (CRI Maestro).

The process for counting positive units is as follows: First, use the ‘Analyze particles’ module in the ‘ImageJ 1.53c’ image processing software to obtain the average fluorescence intensity value for each reaction chamber. Next, plot the mean fluorescence intensity values for all reaction units as a scatter plot using the Origin 2023 data processing software. The fluorescence intensity values of the negative and positive reaction units are distributed in different intervals, with a clear threshold line separating them. Units with a fluorescence intensity distribution above this line are considered positive, while those one below it are negative.

## 5. Results and Discussion

### 5.1. Fast and Simple Sample Loading and Partition

**Figure 4:**
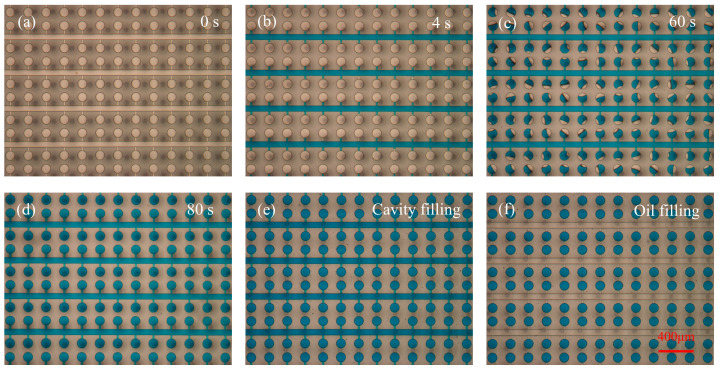
Sample loading and partitioning process of the chip. Initially, the negative pressure was in the outlet of the blank chip (**a**) and the microfluidic channels of the chip were filled immediately (**b**). Then, the PCR solution was drawn into the reaction chambers by vacuuming the cavity above the PCR reaction layer (**c**) and the filling was completed (**d**). Next, the cavity was filled with water via pump suction (**e**). In the end, the PCR solution in the microfluidic channels was replaced with FC40 to isolate the reaction chambers (**f**).

Since the PCR solution is colorless, blue dye was used instead of the PCR solution to investigate the sample loading and partitioning process of the chip. By the pump suction at the outlet (−10 kPa), the sample loaded at the inlet of the chip was perfused through the entire microfluidic channels in less than 5 s (Figure 4a,b). The flow rate of the sample in the microfluidic channel was high due to the direct pressure difference acting on both ends of the channel. Then, negative pressure was applied in the cavity, taking advantage of the favorable permeability of PDMS, to draw the PCR solution into each reaction chamber. The dead-end loading time is influenced by both the pressure exerted within the cavity and the PDMS chip’s thickness. As we analyzed, the larger the negative pressure and the thinner the PDMS film, the shorter the loading time (Figure S3a,b). Given the complexity of the fabrication process, a reaction layer thickness of 400 μm and a pressure differential of 90 kPa were deemed appropriate. Within 80 s, the cavity achieved the sample filling of 60 μm high reaction chambers (Figure 4c,d). Afterwards, the valve of the cavity inlet was opened a little bit and water was drawn into the cavity via pump suction, creating a negative-pressure environment within the chip (Figure 4e). In the end, the fluorinated oil of FC40 was introduced to replace the sample solution in the microfluidic channel, achieving the purpose of isolating the reaction chamber (Figure 4f). The results showed that all the reaction chambers could be completely filled with the blue dye, and a reliable isolation barrier was established with FC40 between the reaction chambers. The entire sample loading and partitioning process of the chip was completed in a total of 2 min.

### 5.2. Water Loss and Bubble Formation of the Chip in the Thermal Reaction

**Figure 5:**
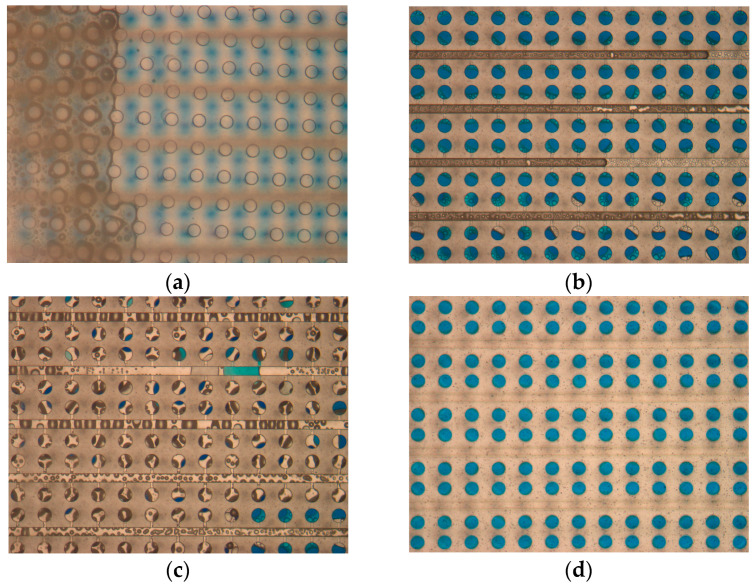
The retention of liquid in the chip after thermal cycling under different negative pressure controls. When the chip, pre-degassed with the negative pressure of 50 kPa, was heated at 95 °C for 20 s, bubbles appeared in the cavity and water gradually receded (**a**). Subsequently, water loss was also observed at the PCR reaction layer (**b**) and gradually worsened (**c**). However, if the negative pressure was increased to 90 kPa, there were no bubbles formed and no liquid loss was observed, even when heated at 95 °C for 30 min (**d**).

Our analysis suggests that as long as the initial pressure in the PDMS is lower than 13 kPa, no bubbles will generate during PCR. To verify this, a microscope-compatible ITO heating platform was used to heat the chip with different internal pressures. When the cavity was pumped at a negative pressure of 50 kPa for 2 min, the water was still able to fill the entire cavity without dead space. A bubble first appeared in the water in the cavity at 95 °C, (Figure 5a). The bubble grew rapidly and the liquid in the cavity broke through the UV adhesive seal at the inlet or outlet and spilled out of the chip; bubble generation was observed in the microfluidic channels and PCR reaction chambers beneath the cavity wherein water was lost (Figure 5b). With continued high temperatures, most of the sample in the PCR reaction chambers disappeared (Figure 5c). However, when the cavity was treated with a negative pressure, at 90 kPa for 2 min, no bubble generation and water reduction were observed on the dPCR chip on a 95 °C heating plate for 30 min (Figure 5d), indicating that bubbles were completely suppressed.

If water vapor does not play a major role, the volume or pressure of gas due to thermal effect increases only 0.23-fold during PCR, and a negative pressure of 50 kPa is sufficient to eliminate the appearance of bubbles. The experimental results indicate that a 50 kPa negative pressure is not enough, and only a more negative pressure can inhibit the generation of bubbles, which indicates that water vapor is the main reason for the emergence of bubbles. The efficient low-pressure system can completely prevent the appearance of bubbles and realize valve-free dPCR.

### 5.3. Digital PCR with the Chip

Since the PCR mixture and blue dye solution are different in physical properties such as ion concentration, viscosity, etc., parallel experiments were conducted using both types of solution. The results showed no significant difference between them, when the same parameters including thermal cycle temperature and degassing pressure were used.

Fluorescent dye was employed as an indicator to assess the uniformity of the chambers by measuring the average fluorescence intensity of each reaction chamber. The fluorescent image of the chip (Figure S4a) was captured and ‘ImageJ 1.53c’ software was utilized to analyze the fluorescence intensities of all the reaction chambers. The results are presented in a histogram (Figure S4b), revealing that the fluorescence intensity of the reaction chambers ranges from 160 to 185. The coefficient of variation (CV) in the fluorescence intensity values among the chambers in the chip is approximately 4.12%, indicating that the reaction chambers were equally filled and the reagents were uniformly partitioned.

**Figure 6:**
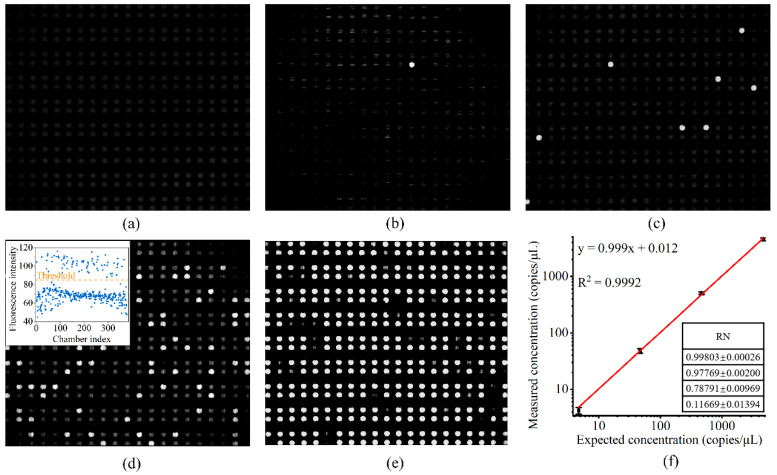
Results of the digital PCR assay for target DNA templates at various concentrations including negative (**a**), 4.7 copies/μL (**b**), 4.7 × 10 copies/μL (**c**), 4.7 × 10^2^ copies/μL (**d**) and 4.7 × 10^3^ copies/μL (**e**). The scatter plot in the inset (**d**) shows that the positive and negative chambers can be clearly classified using a threshold. The correlation plot (**f**) illustrates a linear relationship of 0.9992 between the measured and expected concentrations. RN: ratio of negative wells.

Synthesized templates were utilized to evaluate the quantitative detection capability of the dPCR chip. Representative images of the dPCR chips following PCR amplification are presented in Figure 6. The negative control does not show any positive chambers, while the numbers of positive chambers with the diluted samples obeys the Poisson distribution. The results of the fluorescence intensity distribution of the positive and negative units show a clear distinction between the positive and negative units (the scatter plot in Figure 6d inset). The results of dPCR show that the measured DNA concentration closely matched the expected DNA concentration (R^2^ = 0.9992), which proves the high performance of the dPCR method.

In Figure 6, some reactions are not circular in shape. This result may be due to water in the reaction chambers evaporating into the mesopores inside the PDMS during the PCR reaction. Although the mesopores were mainly filled with water vapor from the cavity layer, a small amount of the PCR solution in the reaction chamber also evaporated into the PDMS, and then a small amount of oil entered the reaction chamber from the branch channels. Therefore, the fluorescence image of a single reaction chamber may not form a complete circle.

## 6. Conclusions

In a digital PCR chip fabricated with PDMS, gas permeability is essential in sample loading. However, gas permeability and solubility result in bubble formation and water loss in the subsequent thermal reaction. To solve these problems, we fabricated a chip with two structure layers and a sealing parylene C shell. The PDMS membrane between the cavity and the reaction chambers was thin and had very good gas permeability, which obviously accelerates sample loading and system degassing. The large size of the cavity could maintain both a lower pressure and saturated steam environment in the chip during thermal cycling. The top surface and side wall of the chip were both coated with parylene C, and inlets and outlets could be easily sealed with UV-glue in several seconds. A favorable negative pressure environment can be established inside the parylene C shell. A small negative pressure (50 kPa) can also load the sample and fill the cavity, but it cannot prevent the emergence of bubbles and the evaporation of the solution in reaction chambers, while a large negative pressure (90 kPa, 2 min) can completely stop the appearance of bubbles. There was no visible loss of water during one hour of the PCR reaction due to the good sealing performance of the shell. A simple-operation and little-time-consuming system that eliminates tube connection for pumps and valves could make the chip suitable to be widely applied in DNA analysis.

## Data Availability

The data presented in this study are available upon request from the corresponding author.

## Supplementary Materials

The following supporting information can be downloaded at https://www.mdpi.com/article/10.3390/bios14030114/s1: Figure S1: Chip manufacturing process; Figure S2: Schematic diagram of ITO heating platform; Figure S3: Sample loading time as a function of applied pressure (a) and PDMS thickness (b); Figure S4: Fluorescent image of the chamber array (a) after loading the fluorescence reagent, and uniformity analysis of the dPCR chip (b); template sequence.

## References

[1] Saiki R.K., Scharf S., Faloona F., Mullis K.B., Horn G.T., Erlich H.A., Arnheim N. (1985). Enzymatic Amplification of β-Globin Genomic Sequences and Restriction Site Analysis for Diagnosis of Sickle Cell Anemia. Science.

[2] Toriello N.M., Liu C.N., Mathies R.A. (2006). Multichannel reverse transcription-polymerase chain reaction microdevice for rapid gene expression and biomarker analysis. Anal. Chem..

[3] Rajagopal A., Yurk D., Shin C., Menge K., Jacky L., Fraser S., Tombrello T.A., Tsongalis G.J. (2019). Significant Expansion of Real-Time PCR Multiplexing with Traditional Chemistries using Amplitude Modulation. Sci. Rep..

[4] Green M.R., Sambrook J. (2019). Nested Polymerase Chain Reaction (PCR). Cold Spring Harb. Protoc..

[5] Heid C.A., Stevens J., Livak K.J., Williams P.M. (1996). Real time quantitative PCR. Genome Res..

[6] Ahrberg C.D., Manz A., Chung B.G. (2016). Polymerase chain reaction in microfluidic devices. Lab. Chip.

[7] Vogelstein B., Kinzler K.W. (1999). Digital PCR. Proc. Natl. Acad. Sci. USA.

[8] Salipante S.J., Jerome K.R. (2020). Digital PCR—An Emerging Technology with Broad Applications in Microbiology. Clin. Chem..

[9] Basu A.S. (2017). Digital Assays Part I: Partitioning Statistics and Digital PCR. SLAS Technol..

[10] Pattanayak P., Singh S.K., Gulati M., Vishwas S., Kapoor B., Chellappan D.K., Anand K., Gupta G., Jha N.K., Gupta P.K. (2021). Microfluidic chips: Recent advances, critical strategies in design, applications and future perspectives. Microfluid. Nanofluidics.

[11] Sackmann E.K., Fulton A.L., Beebe D.J. (2014). The present and future role of microfluidics in biomedical research. Nature.

[12] Sreejith K.R., Ooi C.H., Jin J., Dao D.V., Nguyen N.-T. (2018). Digital polymerase chain reaction technology—Recent advances and future perspectives. Lab. A Chip.

[13] Heyries K.A., Tropini C., Vaninsberghe M., Doolin C., Petriv O.I., Singhal A., Leung K., Hughesman C.B., Hansen C.L. (2011). Megapixel digital PCR. Nat. Methods.

[14] Miranda I., Souza A., Sousa P., Ribeiro J., Castanheira E.M.S., Lima R., Minas G. (2021). Properties and Applications of PDMS for Biomedical Engineering: A Review. J. Funct. Biomater..

[15] McDonald J.C., Whitesides G.M. (2002). Poly(dimethylsiloxane) as a Material for Fabricating Microfluidic Devices. Acc. Chem. Res..

[16] Tian Q., Yu B., Mu Y., Xu Y., Ma C., Zhang T., Jin W., Jin Q. (2015). An integrated temporary negative pressure assisted microfluidic chip for DNA isolation and digital PCR detection. RSC Adv..

[17] Zhou X., Ravichandran G.C., Zhang P., Yang Y., Zeng Y. (2019). A microfluidic alternating-pull–push active digitization method for sample-loss-free digital PCR. Lab. A Chip.

[18] Ning Y., Cui X., Yang C., Jing F., Bian X., Yi L., Li G. (2019). A self-digitization chip integrated with hydration layer for low-cost and robust digital PCR. Anal. Chim. Acta.

[19] Xu G., Si H., Jing F., Sun P., Wu D. (2021). A Self-Priming Microfluidic Chip with Cushion Chambers for Easy Digital PCR. Biosensors.

[20] Lee S.H., Song J., Cho B., Hong S., Hoxha O., Kang T., Kim D., Lee L.P. (2019). Bubble-free rapid microfluidic PCR. Biosens. Bioelectron..

[21] Liu H.-B., Gong H.-Q., Ramalingam N., Jiang Y., Dai C.-C., Hui K.M. (2007). Micro air bubble formation and its control during polymerase chain reaction (PCR) in polydimethylsiloxane (PDMS) microreactors. J. Micromech. Microeng..

[22] Karlsson J.M., Gazin M., Laakso S., Haraldsson T., Malhotra-Kumar S., Mäki M., Goossens H., van der Wijngaart W. (2013). Active liquid degassing in microfluidic systems. Lab. A Chip.

[23] Nakayama T., Hiep H.M., Furui S., Yonezawa Y., Saito M., Takamura Y., Tamiya E. (2010). An optimal design method for preventing air bubbles in high-temperature microfluidic devices. Anal. Bioanal. Chem..

[24] Chen J., Chen D., Xie Y., Chen X., Wang K., Cui D., Du H., Wang Z. (2015). Bubble generation and mechanism in polydimethylsiloxane based polymerase chain reaction chip. Appl. Phys. Lett..

[25] Shin Y.S., Cho K., Lim S.H., Chung S., Park S.J., Chung C., Han D.C., Chang J.K. (2003). PDMS-based micro PCR chip with parylene coating. J. Micromech. Microeng..

[26] Niu Z.Q., Chen W.Y., Shao S.Y., Jia X.Y., Zhang W.P. (2006). DNA amplification on a PDMS–glass hybrid microchip. J. Micromech. Microeng..

[27] Zhu Q., Qiu L., Yu B., Xu Y., Gao Y., Pan T., Tian Q., Song Q., Jin W., Jin Q. (2014). Digital PCR on an integrated self-priming compartmentalization chip. Lab. Chip.

[28] Xu T.G., Wu L., Wang X.F., Zhu X.Y., Bao Y.Y., Cai S.R., Li G., Li X.X. A PDMS-Based Digital PCR Chip with Vacuum Aspiration and Water-Filling Cavity Integrated for Sample Loading and Evaporation Reduction. Proceedings of the 2018 IEEE Micro Electro Mechanical Systems (MEMS).

[29] Ottesen E.A., Hong J.W., Quake S.R., Leadbetter J.R. (2006). Microfluidic digital PCR enables multigene analysis of individual environmental bacteria. Science.

[30] Fu Y., Zhou H., Jia C., Jing F., Jin Q., Zhao J., Li G. (2017). A microfluidic chip based on surfactant-doped polydimethylsiloxane (PDMS) in a sandwich configuration for low-cost and robust digital PCR. Sens. Actuators B Chem..

[31] Xu T.G., Wu L., Wang X.F., Zhu X.Y., Chen J.Z., Yao F.L., Zhou H.B., Li X.X. A Seal-Free Valveless Digital PCR Chip Supported with a High-Pressure Water Circulation System. Proceedings of the 2020 IEEE 33rd International Conference on Micro Electro Mechanical Systems (MEMS).

[32] Ghosal K., Freeman B.D. (2003). Gas separation using polymer membranes: An overview. Polym. Adv. Technol..

[33] Xu L., Lee H., Jetta D., Oh K.W. (2015). Vacuum-driven power-free microfluidics utilizing the gas solubility or permeability of polydimethylsiloxane (PDMS). Lab. A Chip.

