# Supplementary material for: Complete Prevention of Bubbles in a PDMS-Based Digital PCR Chip with a Multifunction Cavity
Source: Biosensors (Basel). 2024 Feb 21;14(3):114. doi: 10.3390/bios14030114 (PMC10968645; doi:10.3390/bios14030114)
Supplement: Supplementary file 1 [file biosensors-14-00114-s001.zip › biosensors-2785878-supplementary.pdf]

Complete prevention of bubbles in PDMS-based digital PCR chip with a multifunction cavity

Shiyuan Gao[1,2,3], TiegangXu [1,2,*], Lei Wu [1,2,*], Xiaoyue Zhu[4] , Xuefeng Wang[1,2] , Ying Chen [1,2], Gang Li[5] and Xinxin Li[1,2,3,*]

Chip manufacturing process

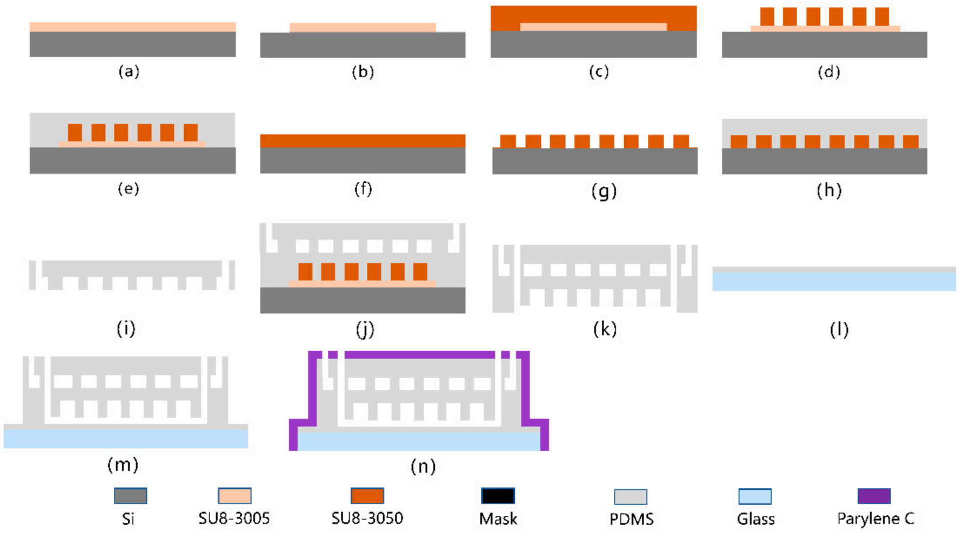

Figure S1. Chip manufacturing process. (a) Spin-coat 10μm thick SU8-3005 photoresist on the wafer surface, (b) Branch channel patterning by photolithography; (c) Spin-coat 60μm thick SU8-3050 photoresist; (d) Main channel and micro-chamber patterning by photolithography; (e) Pour PDMS on the surface of the mold and cure to form a reaction layer; (f) Spin-coat 40μm of SU8-3050 photoresist on the surface of another wafer; (g) Cavity patterning by photolithography; (h) Pour PDMS on the mold and cure to form a cavity layer; (i) Remove the cavity layer from the surface of the mold and punch out the holes; (j) Bond the cavity layer on the surface of the reaction layer; (k) Remove the two bonded PDMS layers from the surface of the mold and punch out the feed holes; (l) Spin-coat a layer of PDMS on the glass surface and cure; (m) Bond the two layers of PDMS to the glass surface; (n) Deposit a Parylene C film on the chip surface.

ITO heating platform

The ITO heating platform was made of a temperature controller, a piece of ITO glass and a Pt100 thermal sensor (Figure S2). The conductive indium tin oxide film on the ITO glass could heat the PCR reaction when a voltage is applied to it. The Pt100 thermal

sensor could detect the surface temperature of the ITO glass in real time. The thermal controller contains a STM32 master chip, which could accurately adjust the temperature of the ITO glass by controlling the duty cycle of the output power according to the input temperature signal.

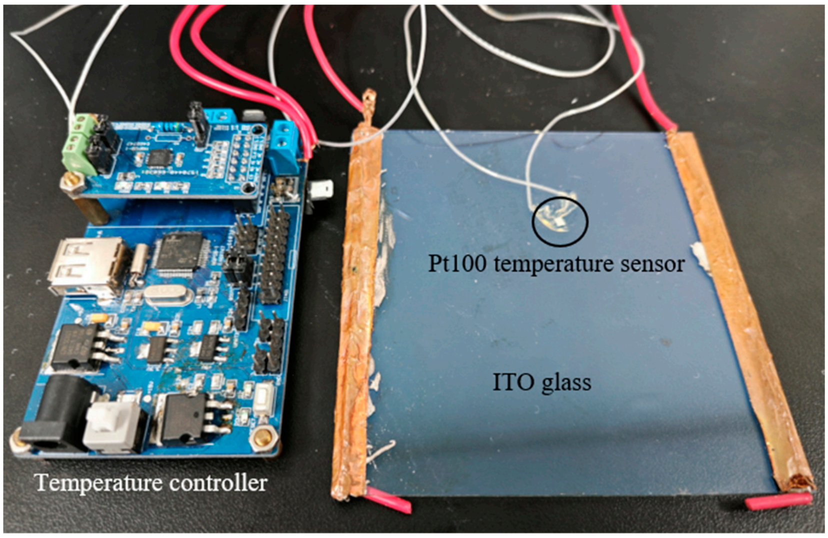

Figure S2. Schematic diagram of ITO heating platform.

Relation of sample loading time with sample loading pressure and PDMS thickness

Sample loading speed is a key indicator of performance for a dPCR chip. Sample laoding pressure and PDMS thickness are two parameters affecting sample loading time. We tested the sample loading time of a 400 μm chip under 100 mbar to 900 mbar pressure (Figure S3a) and the sample loading time under 900 mbar for chip thickness ranging from 300 μm to 800 μm (Figure S3b). The sample loading time decreases with sample loading pressure. Therefore, we chose 900 mbar (maximum of our vacuum pump) as sample loading pressure and found that the sample loading time increased with PDMS thickness. Although membrane with thickness under 400 μm is easily torn during demolding, the sample loading time will be elongated if the membrane is too thick. Therefore, we chose an optimized chip thickness to be 400 μm. Under 900 kpa pressure, the sample loading time in the 400 μm thick chip was only 80 s. Therefore, this chip enables sample loading in short amount of time.

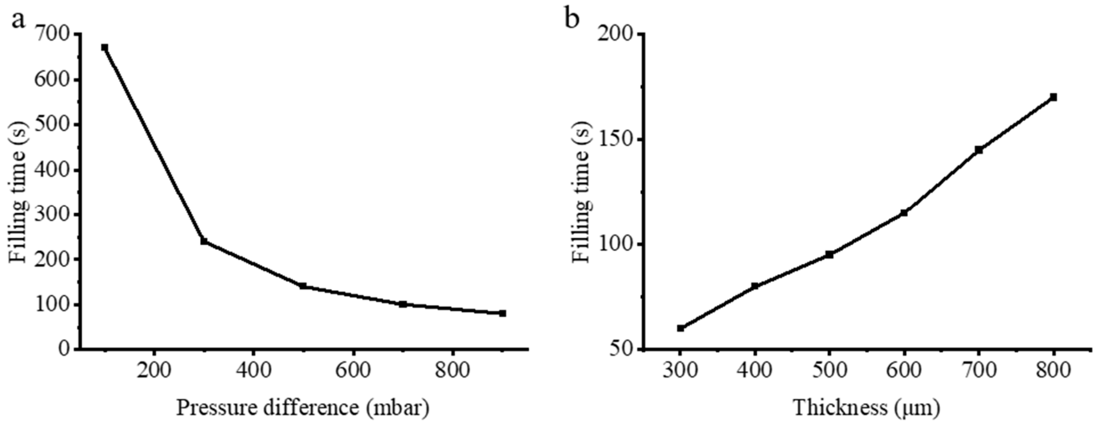

Figure S3. Sample loading time as a function of applied pressure (a) and PDMS thickness (b).

Uniform dispersion of the sample

Digital PCR technology utilize the fluorescent detection technology and Poisson distribution statistics to quantify the absolute number of DNA target in sample solution. The accuracy of the dPCR will be heavily influenced by the volume variation in the reaction chambers. Rhodamine B were used to examine the uniformity of fluorescent intensity. A solution of 10μM Rhodamine B was loaded into the PCR chip and partitioned with FC-40. Figure S4a shows the fluorescent image after dye loading. Figure S4b shows the frequency of fluorescent intensity in each reaction chambers. The relative standard deviation is about 4.12%. Results showed that the fluorescent intensity is uniform among different reaction chambers, proving that the sample volumes in each reaction chamber are about the same.

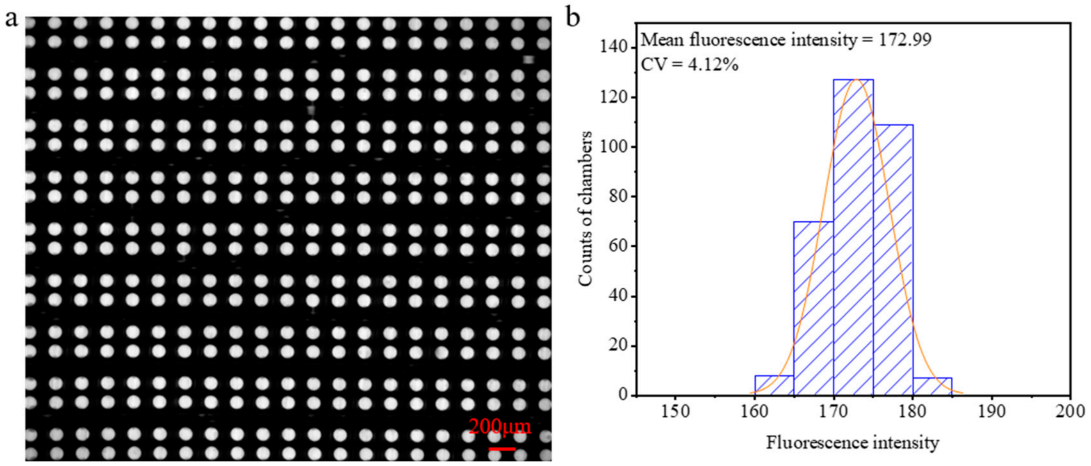

Figure S4. Fluorescent image of the chambers array (a) after loading fluorescence reagent, and uniformity analysis of the dPCR chip (b).

Template sequence

5-GGGACTCTGGATCCCAGAAGGTGAGAAAGTTAAAATTCCCGTCGCTATCAAGGAATTAAG

AGAAGCAACATCTCCGAAAGCCAACAAGGAAATCCTCGATGTGAGTTTCTGCTTTGCTGTG

TGGGGGTCCATGGCTCTGAACCTCAGGCCCACCTTTTCTCATGTCTGGCAGCTGCTCTGCTC

TAGACCCTGCTCATCTCCACATCCTAAATGT-3
